# A rapid and practical technique for real-time monitoring of biomolecular interactions using mechanical responses of macromolecules

**DOI:** 10.1038/srep28001

**Published:** 2016-06-16

**Authors:** Mehmet C. Tarhan, Nicolas Lafitte, Yannick Tauran, Laurent Jalabert, Momoko Kumemura, Grégoire Perret, Beomjoon Kim, Anthony W. Coleman, Hiroyuki Fujita, Dominique Collard

**Affiliations:** 1 CIRMM, Institute of Industrial Science, The University of Tokyo, Tokyo, Japan; 2 LIMMS/CNRS-IIS, UMI 2820, Institute of Industrial Science, The University of Tokyo, Tokyo, Japan; 3 LMI, UMR 5615, University of Lyon 1, Villeurbanne, France; 4 IEMN, UMR 8520, Villeneuve d’Ascq, France

## Abstract

Monitoring biological reactions using the mechanical response of macromolecules is an alternative approach to immunoassays for providing real-time information about the underlying molecular mechanisms. Although force spectroscopy techniques, *e.g.* AFM and optical tweezers, perform precise molecular measurements at the single molecule level, sophisticated operation prevent their intensive use for systematic biosensing. Exploiting the biomechanical assay concept, we used micro-electro mechanical systems (MEMS) to develop a rapid platform for monitoring bio/chemical interactions of bio macromolecules, *e.g.* DNA, using their mechanical properties. The MEMS device provided real-time monitoring of reaction dynamics without any surface or molecular modifications. A microfluidic device with a side opening was fabricated for the optimal performance of the MEMS device to operate at the air-liquid interface for performing bioassays in liquid while actuating/sensing in air. The minimal immersion of the MEMS device in the channel provided long-term measurement stability (>10 h). Importantly, the method allowed monitoring effects of multiple solutions on the same macromolecule bundle (demonstrated with DNA bundles) without compromising the reproducibility. We monitored two different types of effects on the mechanical responses of DNA bundles (stiffness and viscous losses) exposed to pH changes (2.1 to 4.8) and different Ag^+^ concentrations (1 μM to 0.1 M).

Mechanical properties of molecules are used to investigate a wide range of biological and chemical mechanisms varying from nucleic acid conformation to enzymatic reaction kinetics and from cell manipulation to motor protein operation^1^. Unlike some of the other well-established methods that are often limited to smaller sized molecules, *e.g.* nuclear magnetic resonance (NMR)^2^ and surface plasmon resonance (SPR)^3^, force spectroscopy techniques, *e.g.* optical tweezers^4^,^5^, magnetic tweezers^6^,^7^,^8^ and AFM^9^,^10^, provide sensitive measurements on mechanical properties spanning six orders of magnitude in length (10^−10^–10^−4^ m) including macromolecules^1^,^11^,^12^. These techniques provide information at the single molecule level to reveal novel properties of various different molecules. However, low throughput, surface modification, high-level operational skills and complicated calibration/setup procedures exclude these techniques for rapid routine tests that are essential to provide statistically significant biomechanical information especially for clinical studies^13^. As a result, a rapid, practical, time- and cost-efficient, automated and preferably portable method is beneficial as a complementary or alternative method to the conventional techniques to be employed even by non-specialists.

MEMS technology presents certain advantages to cover the mentioned requirements for routinely performed assays on mechanical responses of macromolecules. MEMS, capable of conducting real-time mechanical sensing with electrical readouts associated with low noise and high stability measurements^14^, allows low cost manufacturing (due to batch processing) and microfluidic integration to perform bioassays. A compact MEMS device (with specific regions for actuation, detection and reaction) integrated with microfluidics provides label- and substrate-free real-time monitoring and manipulation of bio macromolecules. However, operating MEMS electrostatic actuation in liquid is limited to solutions with low-ion concentration as applied voltage causes the formation of double layers that hinder electrostatic force generation^15^. Although large stroke actuation of electrically isolated structures can be monitored in liquid^16^, high damping degrades the sensing capability. Consequently, MEMS devices perform very poorly in liquid limiting their use for bioassays.

Optimal MEMS functioning requires being in air while bioassays have to be performed in liquid. Therefore, we propose a design using tweezers with sharp protruding tips (Fig. 1a) to provide minimal liquid-immersion for a stable, sensitive and low-noise system by keeping the actuating and sensing elements of the device working in air. One of the benefits of the proposed method is the possibility of performing both static and dynamic measurements. Unlike aforementioned conventional methods that are either not capable or showing extremely limited performance of dynamic measurements in liquid, the proposed technique measures mechanical properties such as stiffness and viscous losses by monitoring the frequency response of the molecules between the tips of the tweezers. In contrast to large changes in the frequency response observed when the molecules becoming stiffer due drying in air, *e.g.* DNA^17^ (Fig. 1b), measurements in liquid show smaller changes in rigidity and losses as the molecules remain softer^18^ and thus, require an extremely stable and sensitive system. Here, we overcome these challenges and propose a MEMS-based method for fast dynamic measurements to monitor mechanical responses of bio macromolecules exposed to different solutions.

## Results

**Figure 1:**
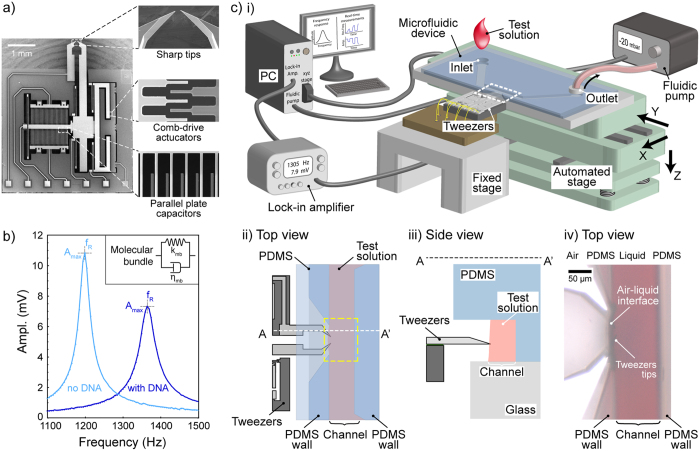
(**a**) SEM images of MEMS tweezers. One of the sharp tips (right tip) of the tweezers was actuated using comb-drive actuators and sensed with integrated differential capacitive sensor. (**b**) Capturing a DNA bundle between the tips of the tweezers caused an increase in f_R_ and a decrease in A_max_. (**c**) Setup of the proposed method. ((**c**)i) Tips of the tweezers entered the side opening of a microfluidic device consisted of a PDMS slab placed on a cover slip. Tweezers were mechanically driven and sensed by a lock-in-amplifier. A pressure pump controlled the flow in the channel of the microfluidic device enabling multi-solution testing. A LabVIEW program was used to run the experiments controlling all equipment. ((**c**)ii) Top view illustration of the white-dashed rectangle in ((**c**)i) is shown. Only the tips of the tweezers entered the channel via the side opening. ((**c**)iii) Side view illustration of the white-dashed line (A-A’) in ((**c**)ii). The PDMS rim was used for the positioning process. ((**c**)iv) Top view image (by Keyence VHS-500) of the tweezers tips inserted into channel (filled with red ink) via the side opening. The corresponding area is illustrated with yellow-dashed rectangle in ((**c**)ii).

The proposed technique involved two devices: MEMS tweezers and a microfluidic device (see Methods). Micro machining techniques were used to fabricate the MEMS tweezers with sharp protruding tips actuated by electrostatic comb-drive actuator that was connected to a capacitive displacement sensor (Fig. 1a). The microfluidics device consisted of a microfluidic channel with an inlet, an outlet and another opening on the side for inserting the tips of MEMS tweezers. The air-liquid interface remained stable due to the surface tension influenced by the shape and materials around it. The microfluidic device was positioned using a computer-controlled XYZ stage with nanometric accuracy. A pressure pump working in withdraw mode was connected to the outlet to generate a solution injection flow via the inlet allowing multiple solutions to be tested successively on the same molecules between the tips. A lock-in-amplifier drove MEMS tweezers and monitored the frequency response to detect changes in the resonance frequency (f_R_) and the maximum amplitude (A_max_, Fig. 1c, see Methods).

Fast and reliable positioning of the tweezers relative to the air-liquid interface at the side opening was successfully achieved. This crucial step guarantied the same interface conditions for each experiment as an essential setup requirement to perform high-sensitivity rapid testing. The molecular sensing element of MEMS tweezers were used as a precise self-positioning tool by monitoring sudden changes in the frequency response on penetration of the air-liquid interface. Using a 3-step protocol for precise positioning (see Methods, Supplementary Fig. 1), we could map the air-liquid interface to decide on the suitable immersion height. Air-liquid interface position above z = 30 μm (from the glass surface) was within a 0.5-μm range (for h = 120 μm, Supplementary Fig. 2). Closer to the glass surface, the interface was positioned further away from the channel (>2 μm) due to the hydrophilic characteristics of the glass surface.

**Figure 2:**
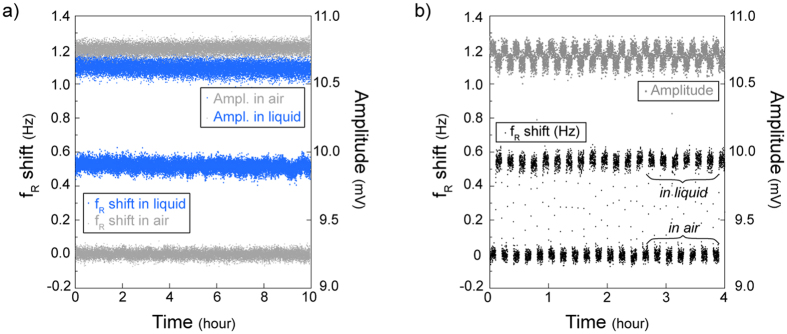
(**a**) Real-time measurements for more than 10 hours demonstrated the stability of the system. The measurements were displayed as f_R_ shift between the air and liquid conditions for each experiment. The result shows an f_R_ shift of 0.48 + 0.03 Hz and an A_max_ of 10.62 + 0.04 mV (mean + std. dev.) with respect to the in-air result (0.00 + 0.02 Hz, 10.76 + 0.03 mV). (**b**) Repeated insertion and removal cycles showed the same f_R_ shift indicating the stability and repeatability of the insertion protocol. 6 minutes cycles of in-air and in-liquid measurements were performed for 4 h.

As detecting changes only in the molecules between the tips required a very stable air-liquid interface, we first checked the stability of the interface to guarantee the performance for long measurements and repeated liquid insertions. The stability of the interface was tested using bare tweezers (immersed 5 μm in deionized water) with 10-hour real-time measurement in the temperature-controlled environment (Fig. 2a). Tweezers in liquid resulted in an f_R_ shift of 0.48 + 0.03 Hz and an A_max_ of 10.62 + 0.04 mV (mean + std. dev.) with respect to the in-air result (0.00 + 0.02 Hz, 10.76 + 0.03 mV). In addition, the stability of the system was tested for consecutive measurements in air (50 μm away from the interface) and in liquid (with an immersion of 5 μm). Stability of the successive 6-min measurements showed that the interface was not affected by repeated tweezers insertion cycles (Fig. 2b). The induced mass due to liquid immersion, and the surface tension at the air-liquid interface inversely affected the f_R_ shift. Due to the small size of the side opening and negligible effect of 5-μm-liquid immersion compared to the moving part (i.e. the combination of the actuating arm, common electrode of the differential sensor and the grounded combs of the actuator, Fig. 1a), high surface tension dominated causing an increase in f_R_ (Supplementary Fig. 3).

**Figure 3:**
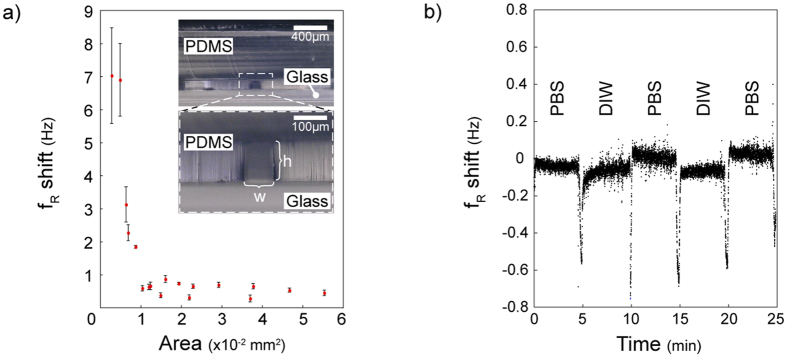
(**a**) The side opening showed stable results for window area >0.01 mm^2^. Inset photos show side view of the channel side opening with a width *w* and a height *h*. Error bars correspond to standard deviations. (**b**) Solution inside the channel could be exchanged using the pressure pump in the vacuum mode. 20 s periods of liquid withdrawn (lower spikes) followed by no-flow periods. For each 5-min cycle, the liquid inside the channel was changed between deionized water (DIW) and phosphate buffered saline (PBS) solution.

There were 3 setup parameters affecting the stability: (i) immersion depth, (ii) immersion height and (iii) side opening dimension (Fig. 3a inset). Excessive immersion depth (>12 μm for w = 108 μm; Supplementary Fig. 4) caused instability due to the capillary effect between the arms of the tweezers and the PDMS walls. Similarly, placing tweezers too close to the glass surface (<30 μm for h = 115 μm; Supplementary Fig. 5) affected the stability due to the capillary effect between the bottom of the tweezers and the glass surface for different opening dimensions (Supplementary Figs 4–6). Devices with a side opening >0.01 mm^2^ (w > 110 μm, h > 90 μm) showed high stability in the experiments (Fig. 3a, Supplementary Figs 7 and 8).

Exchanging solutions inside the channel enabled the key feature of testing multiple-solutions on the same macromolecule bundle. The pressure pump was used in withdrawal mode to generate a flow in the channel (see Methods). Applying different pressure levels resulted in different flow speeds without compromising stability (Supplementary Fig. 9). Decreasing the pressure in the pump (−30 mbar for 20 s) withdrew the liquid into the channel (*e.g.* DI water) allowing replacement with another solution (*e.g.* phosphate buffered saline, PBS) via the inlet port as demonstrated with 5-min exchange cycles. A flow for liquid exchange was induced only at the last 20 s of the 5-min periods (Fig. 3b). Solutions having different viscosity and/or surface tension caused small f_R_ shifts (in the mHz order).

**Figure 4:**
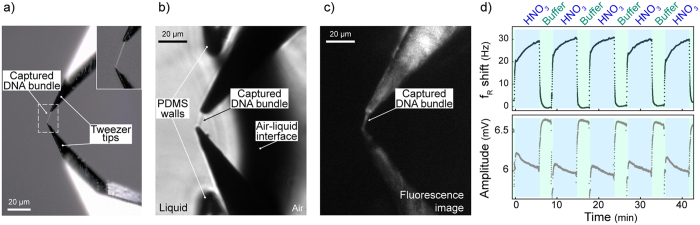
(**a**) A DNA bundle was captured between the tips of the fabricated tweezers. The inset image corresponds to the white-dashed rectangle. (**b**) To demonstrate the successful insertion of the DNA bundle, a labeled DNA was captured and visualized inside the channel on an inverted microscope stage (Olympus IX71) with visual light. (**c**) Fluorescence image showed the captured DNA between the tips of the tweezers inside the channel (at the same position as (**b**)). For better visualization, tips of the tweezers were inserted deep in the channel. (**d**) Effect of acid on the mechanical properties of the DNA bundle was reversible. Tris buffer (pH 6.8) followed HNO_3_ solution (pH 4.1) injection (6 min of HNO_3_ and 3 min of buffer cycles). HNO_3_ solution increased f_R_ and decreased A_max_ of the tweezers and Tris buffer returned them back to the initial values.

We captured DNA as the macromolecule for testing the performance of the proposed method to monitor the mechanical effects of bio/chemical reactions (see Methods). Dielectrophoresis (DEP)-assisted lateral combing was performed to capture a λ-phage DNA bundle bridging between tweezers tips (Fig. 4a). A drop (5 μl) of λ-phage DNA solution was placed on the PDMS device. Using the automated XYZ stage, tweezers tips were placed inside the droplet while applying an AC voltage (1 MHz, 3.2–4.8 V_p-p_) between the tweezers tips (gap: 8–12 μm). Then, lateral motion of the microfluidic device (Supplementary Fig. 10) allowed one of the tips to leave the droplet with a DNA bundle drawn out. As the second tip left the droplet, the DNA bundle was captured between the two tips of the tweezers (Supplementary Mov. 1).

Captured DNA bundle was penetrated through the air-liquid interface into the channel within seconds, using the automated process. DNA attachment to the aluminum-coated surface was strong enough to overcome the effect of the surface tension during insertion. To demonstrate the success of this step, YOYO1-labeled DNA was captured between the tips and inserted into the channel (Fig. 4b,c).

The effect of pH on DNA is known to be reversible^19^. To test the effect of pH on the mechanical properties of the DNA bundle, solution inside the channel was changed repeatedly applying acid/buffer cycles. Each cycle consisted of an HNO_3_ solution (pH 4.1) followed by (0.1 mM) Tris HCl solution (pH 6.8). After 20 s of flow, 6 min of no-flow incubation period (3 min for the buffer solution) was performed to monitor the effects of the solutions. Decreasing pH increased f_R_ and decreased A_max_ (Fig. 4d). Inversely, the buffer returned the DNA to its initial condition. Repeated cycles indicated the reversible effect of pH on the mechanical properties of the DNA bundle. Moreover, similar frequency responses during the repeated cycles proved that the flow induced during liquid exchange (parallel to the DNA bundle orientation) did not destroy the DNA bundle.

**Figure 5:**
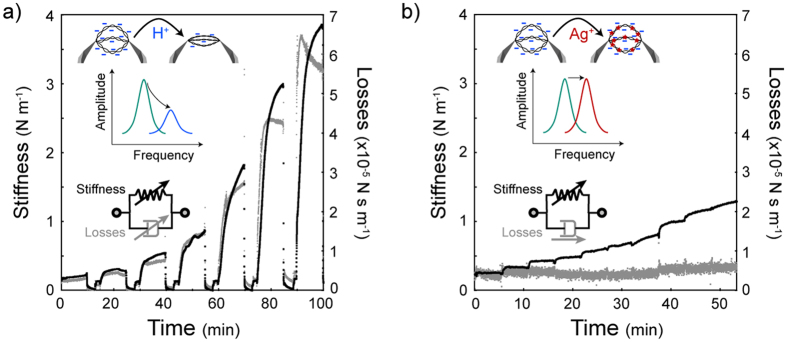
(**a**) Real-time measurements testing the effect of pH (HNO_3_ solution, pH 4.8 to 2.1) on a DNA bundle was performed. Stiffness and viscous losses values were measured using f_R_ shift, A_max_ and mechanical properties of tweezers. Decreasing pH caused an increase in the stiffness and the viscous losses of the bundle. In between each different pH measurements (for 10 min), incubation in Tris HCl solution (pH 6.8) was performed for 5 min (3.5 min of 10 mM Tris HCl solution followed by 1.5 min of 10 μM Tris HCl solution). (**b**) Real-time measurements testing the effect of Ag^+^ concentration (10^−6^ M to 10^−1^ M) was performed on a different DNA bundle. Increasing Ag^+^ concentration resulted in an increase in the stiffness of the bundle. However, unlike the pH case, the viscous losses remained constant.

Different bundles resulted in different amounts of change in f_R_ and A_max_ depending on the amount of captured DNA^20^ and the tweezers characteristics. For better characterization of the bundle, we derived stiffness and viscous losses of the bundle to remove the individual tweezers effect from the results using damped harmonic oscillator model^21^. In this way, we examined the effect of decreasing pH (4.8 to 2.1 in 7 steps using HNO_3_) on the DNA bundle in real-time during 10 min of incubation time (Fig. 5a). Tris buffer solution was applied in between each step. To minimize the effect of remaining Tris HCl solution in the channel for relatively higher pH conditions, we applied buffer in two steps: **(i)** High concentration (10 mM) Tris HCl for 3.5 min to return DNA bundle to initial condition which was followed by **(ii)** low concentration (10 μM) Tris HCl for 1.5 min to minimize the effect of the remaining buffer in the channel before introducing HNO_3_ solutions. The results showed an increase in the stiffness and viscous losses with decreasing pH level although the control (no DNA case) showed no such increase (Supplementary Fig. 11a).

As Ag^+^ attaches to the DNA base pairs^22^, a similar approach was taken for investigating the Ag^+^ effect on the mechanical properties of a DNA bundle. Unlike the pH case, a reversible effect was not detected for Ag^+^. Therefore, we performed real-time monitoring of the effect of increasing Ag^+^ concentration (1 μM to 0.1 M of AgNO_3_) in 10 consecutive steps of 6 min incubation time including a 20-s solution exchange period (Fig. 5b). The results showed an increase in the stiffness (unlike the no-DNA control case, Supplementary Fig. 11b) but no change in the viscous losses with increasing Ag^+^ concentration.

**Figure 6:**
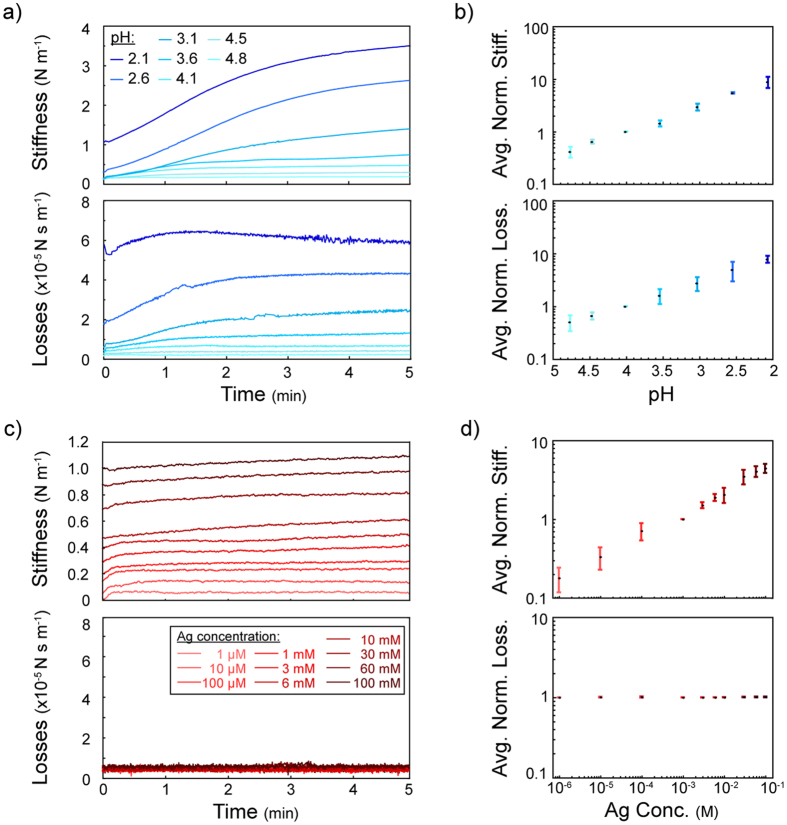
(**a**) Different pH levels affected stiffness and viscous losses at different levels. (**b**) DNA bundles showed similar characteristics under different pH levels. Results of 3 different bundles were normalized according to their response at pH 4.1. (**c**) Increasing Ag^+^ concentration resulted in an increase in the stiffness. However, the viscous losses stayed constant even at higher Ag^+^ concentrations. (**d**) Results of 4 different bundles were normalized according to their response at 1 mM Ag^+^ concentration. All error bars correspond to standard deviations.

The increase in the stiffness and viscous losses for the decreasing pH was in the linear regime (Fig. 6a). Even though the effect of different tweezers was eliminated using bundle stiffness and viscous losses values, the effect of the captured bundle size still existed (Supplementary Fig. 12). Bigger bundles resulted in higher stiffness and viscous losses^20^. We averaged the stiffness and viscous losses values (between t = 4 min and t = 5 min) for each pH level and normalized the pH effect on 3 different bundles using their responses at pH 4.1 for better comparison (Fig. 6b).

Increasing Ag^+^ concentration caused an increase in the stiffness. However, unlike the case of pH, regardless of the increasing Ag^+^ concentration, viscous losses remained constant (Fig. 6c, Supplementary Fig. 13). Similar to the pH case, although the effect of tweezers could be eliminated, the effect of the bundle size still existed. We averaged the stiffness and viscous losses values (between t = 4 min and t = 5 min) for each Ag^+^ concentration level and normalized 4 different bundles using their responses in (1 mM) AgNO_3_ solution (Fig. 6d).

The effect of Ag^+^ on DNA stiffness was selective and specific even in a mixture of metal ions (Supplementary Fig. 14). We compared 3 different solutions: (a) metal-ion-mixture solution (1 mM of each: Na^+^, K^+^, Mg^2+^, Ca^2+^ and Co^2+^), (b) Ag^+^ solution (1 mM) and (c) Ag^+^-added metal-ion-mixture solution (1 mM of each: Na^+^, K^+^, Mg^2+^, Ca^2+^, Co^2+^ and Ag^+^). Although the pH values of all 3 solutions were almost identical (4.8, 4.9 and 4.8 respectively), the effect on DNA bundles was different depending on the presence of Ag^+^ in the solution. Ag^+^ solution (solution b) and Ag^+^-added metal-ion-mixture solution (solution c) were tested on different DNA bundles (after exposing bundles to metal-ion-mixture solutions, *i.e.* solution a). For both cases (solutions b and c), f_R_ shift was roughly ~4 times of the shift caused by metal-ion-mixture solution (solution a) because of the selective and specific effect of Ag^+^.

## Discussion

In the experiments, we observed two different effects on DNA bundles. Lower pH (with higher amount of H^+^ in the solution) resulted in protonation of DNA due to a decrease in the negative charges along the phosphate backbone although this may also be associated with a conformational shift^23^,^24^. At low pH levels, base pair unstacking occurs^25^ which might be the reason of the overshoots seen in the viscous losses behavior (Fig. 5a). Even at pH 2.1, the repeated acid/buffer cycles resulted in similar responses (Supplementary Fig. 15). This suggests that although, under our conditions, the DNA molecules were possibly unstacked, the phosphate backbones were still intact between the tips of the tweezers. As the amount of charges was decreased, DNA molecules started repelling each other less, becoming shorter^26^ and forming a tighter bundle with higher intermolecular interaction (Supplementary Fig. 16) in addition to a change in the intramolecular spacing. Tighter bundles resulted in higher concentrations of DNA that caused higher viscous losses^27^,^28^. As a result, both the stiffness and the viscous losses increased with decreasing pH, *i.e.* higher protonation. On the other hand, Ag^+^ intercalated in between the base pairs^22^ causing an increase in stiffness. However, as molecular surface charges and the correlated bundle tightness did not alter, we did not observe any change in viscous losses (Fig. 5 insets).

One of the merits of the demonstrated MEMS-based technique was performing dynamic measurement effectively. Other techniques, *e.g.* AFM, optical and magnetic tweezers, suffer from damping effect in liquid and thus, show poor dynamic measurement performance. As the demonstrated technique minimized the liquid immersion with just the tips of the tweezers while obtaining a stable air-liquid interface with the microfluidic device, the Q-factor of the tweezers in air and in liquid (with no macromolecule between the tips) was almost identical (∆Q-factor <2%). Therefore, the changes in the Q-factor measurements were only due to the changes in the mechanical properties of the macromolecules as demonstrated by two different effects observed on DNA bundles. As a result, the technique could be used for both the dynamic and static measurements in liquid.

The demonstrated experiments have shown real-time monitoring of multiple solutions on the same DNA bundle. Due to the highly stable characteristics of DNA molecules, much longer observations testing tens of solutions (>3 h and >40 times of solution exchange; Supplementary Fig. 17) could be performed without compromising the stability. Regardless of the length of the experiments, preparation (including device setup, liquid injection, tweezers positioning and DNA capturing) required not more than 15 min (see Methods). The necessary time for liquid injection differed depending on the dimensions of the channel and the length of the tubing connected to the outlet. Initial filling of the lower channels (h < 60 μm) was slower due to pronounced effect of PDMS hydrophobicity.

The proposed method is not limited to the demonstrated chemical reactions or attachments. We can apply the method to any kind of interactions producing biomechanical effect on the handled molecules. Therefore, the real-time monitoring can be extended to enzymatic reactions, hybridization, melting and polymerization.

Although only DNA was demonstrated in the experiments, the mechanical characterization can be extended to nano tubes, polymer fibers, *e.g.* polylactic acid, and other filamentous proteins, *e.g.* microtubules (Supplementary Fig. 18), stress fibers and fibronectin. Adjusting the gap between the tips of tweezers, fibers with a wide range of length (from sub-micron to hundreds of micrometers) can be tested in multiple solutions. Moreover, changing the tip geometry, mechanical characterization of single cells or even tissue samples can be performed. Therefore, the system can be adopted by nano technology and material science in addition to biophysical studies. As the mechanical properties of the cells potentially reflect the state on their health^29^, cancer studies^30^, as well, can benefit from this technique as a part of routine clinical examinations.

The proposed method provided label- and substrate-free real-time monitoring of biomolecular interactions. Unlike most existing methods, no surface modification nor complicated characterization/preparation steps were required because the main components of the experiment (*i.e.* actuation, detection and experimental regions) were integrated in the MEMS device. Moreover, as almost all the steps were controlled by software, minimal operational skills were required for proper functioning. Consequently, fast setup, simple operation and portable characteristics enabled the proposed method to perform routine tests on macromolecules.

## Methods

### Fabrication

**MEMS tweezers** were fabricated by standard micro machining process flow from <100> oriented Silicon-On-Insulator (SOI) wafer, with double sides Deep Reactive Ion Etching (DRIE) and vapor HF releasing of the movable structures. The sharp tips engineering included a local oxidation of silicon (LOCOS) and wet anisotropic etching for revealing <111> crystallographic planes. The spring structures and inertia center of the tweezers were carefully designed to obtain a parallel motion both of the movable tip and capacitive sensing structures providing accurate sensing^31^. Protruding tips with an angle of 130° allowed tweezers to operate inside the microfluidic device with minimum immersion. Based on a generic process flow, the final gap could be controlled up to several tenths of micron by a simple single-step dry etching time adjustment, depending on the targeted trapping application (*e.g.* DNA, cells). As experiments were performed on 16-μm-long λ-phage DNA, tweezers with a gap of 8–12 μm were used.

**Microfluidic device** was a PDMS slab placed on a cover slip. A microchannel was molded in the PDMS slab with an inlet, an outlet and a side opening. SU8 lithography was performed to obtain the structures for PDMS molding. Devices having different channel height (varying from ~50 μm to ~250 μm) and side openings width (varying from ~50 μm to ~200 μm) were fabricated with a channel width of 100 μm. We opened holes in the PDMS slab to form the inlet and the outlet by using 1-mm and 0.5-mm width punchers respectively. We connected the outlet to the pressure pump with peek tubing.

The PDMS slab was placed on a cover slip manually using a microscope (with 20x magnification). The edge of the cover slip was aligned with the PDMS wall (width of 300 μm) keeping the brimming part of the PDMS slab (>500 μm) hanging out of the cover slip (Fig. 1c).

### Tweezers working principle

The mechanical working principle and design parameters of tweezers were published elsewhere^32^,^33^. Using harmonic analysis, the main mechanical resonance of the system was monitored in real-time. An AC signal (1 V_rms_) was applied for actuation. The motion was sensed through measurements of two electrical currents from the integrated sensor. The sensor consisted of three parallel plate electrodes named as C_0_, C_1_ and C_2_. C_1_ and C_2_ were fixed electrodes. The central electrode C_0_ was mechanically connected to the actuating tip, forming two identical capacitances when there were no actuation or external forces. When C_0_ was polarized with a constant voltage V_0_ (3 V) and the fixed electrodes were kept grounded, the tip motion changing the gaps between the parallel plates created dynamics currents (i_c1_ and i_c2_) that were collected from C_1_ and C_2_. To measure these pico-ampere capacitive currents, two low-noise current-to-voltage (A/V) preamplifiers (Signal Recovery, model 5182) converted the currents i_c1_ and i_c2_ into voltages, respectively V_1_ and V_2_. The low input impedance of the preamplifier (virtual ground, *i.e.* 0 V) ensured an accurate current conversion with a gain of 10^8^. Finally, a lock-in amplifier (Signal Recovery, AMETEK 7270 DSP) provided a magnitude-phase measurement of the differential inputs (V_1_-V_2_) at the reference frequency. We performed the measurements for each frequency to provide the resonance response of the device (Supplementary Fig. 19a).

Tweezers were designed to perform lateral in-plane motion at their first resonance mode. The damped harmonic oscillator^21^ provided an accurate model for this mode (Supplementary Fig. 19b). In the experiments, the resonance characteristics of the tweezers with immersed tips were recorded just before the molecular assay and f_R_ (resonance frequency), Q (quality factor) and A_max_/Q (chain gain) were extracted by least square method. The tweezers stiffness, *k*, and viscous looses, *η*, were calculated by equations (1) and (2) (with *k*_*mb*_ = 0 and *η*_*mb*_ = 0). Using typical tweezers with a mass (*M*) of 360 μg in Supplementary Fig. 19a, we could extract the stiffness and losses values as k = 20.3 N m^−1^ and *η* *=* 54.9 μN s^−1^.

During the molecular assays, a LabVIEW program-controlled phase lock loop (PLL) kept the tweezers at resonance using the phase of the motion. The real time resonance frequency, (f_R_(t)), maximum amplitude (A_max_(t)) and quality factor (Q(t) using the chain gain A_max_/Q) allowed real time calculations of the changes in the molecular bundle characteristics, *i.e.* stiffness (*k*_*mb*_) and viscous losses (*η*_*mb*_), using equations (1) and (2).


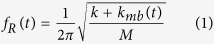



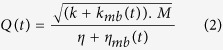


### Setup

Experiments were performed in a temperature-controlled box. The box included a fixed stage for tweezers, an XYZ stage (SLC-1720S-STU-XYZ, SmarAct GmbH) for microfluidic device, a computer-controlled heater (PS25100-24/12, Nippon-heater Co., Ltd.), a humidity/temperature sensor (TSP01/TSP-TH, Thorlabs, Inc.) and interface (*e.g.* electrical and fluidic connections) for accessing to necessary equipment. Although optical visualization was not mandatory for proper functioning of the method, we monitored the experiments with a non-inverted microscope (Keyence VHX-500) except for the cases when Supplementary movies were shot and fluorescence images were taken using an EMCCD camera (Photometrics CascadeII 512) on an inverted microscope (Olympus IX71) stage.

Sensor electrodes, C_1_ and C_2_, of the tweezers were connected to the lock-in amplifier via two low-noise pre amplifiers (Signal Recovery, model 5182). C_0_ was connected to a voltage source for DC polarization. The lock-in amplifier output was driving the actuator of the tweezers at the necessary frequency. The tips of the tweezers were connected to a function generator (Agilent 33220A) for applying an AC voltage during DEP-assisted DNA capturing. All peripheral equipment (the lock-in amplifier, the function generator, the XYZ-stage and the humidity/temperature sensor) was directly connected to the computer. The heater, on the other hand, was driven by a current source that was also connected to the computer.

Characterization experiments were performed with a 100-ms time resolution (Figs 2 and 3). Though we presented only raw data in the figures (except from Supplementary Fig. 20 for demonstration purposes), a smoothing algorithm could be used to monitor smaller changes. For instance, although noticing the temperature effect on the frequency response was difficult with the raw data (Fig. 2a), result clearly showed the correlation between the temperature and f_R_ after smoothing. Temperature change of 0.2 °C inside the box caused ~0.02 Hz oscillations in f_R_ (Supplementary Fig. 20).

To test the effect of different solutions, we injected liquid to the channel via the inlet of the microfluidic device. The outlet was connected to a pressure pump (Elveflow AF1 Dual) for generating flow when necessary. The flow was detected with a microfluidic flow sensor (Elveflow MFS). To minimize the effect of evaporation for long experiments (Fig. 2), a syringe-pump was utilized to feed the inlet automatically.

### Tweezers insertion protocol

The motion of the microfluidic device (on the XYZ stage) was controlled with a PC for precise relative positioning (Supplementary Fig. 1). To start the insertion protocol, the PDMS rim was placed over the tweezers. (i) The first step was to detect the relative position of the PDMS layer (top of the channel) and the tweezers. The developed LabVIEW software ran the XYZ stage (0.1 μm steps with a speed of 100 μm s^−1^) while monitoring f_R_. When the PDMS layer touched the tweezers, the lock-in-amplifier detected the sudden change in the frequency response (amplitude and phase) and stopped the XYZ stage. The microfluidic device was then moved 30 μm away from tweezers for safe insertion between the PDMS and glass layers. (ii) After moving the microfluidic device toward the tweezers, the second step was to detect the top and bottom level position of the channel (PDMS and glass levels). The same detection technique was used: sudden changes in the frequency response. Finally, the device was placed depending on the immersion height at which the experiments were to be performed. (iii) The third step was to detect the air-liquid interface. Moving the microfluidic device toward the tweezers, once more, a sudden change in the frequency response was detected instantly when the tweezers touched the interface. Then, the device was moved back to a safe position (50 μm away from the tweezers; Supplementary Mov. 2).

All the microfluidic devices used in the experiments satisfied the conditions for stable detection (*i.e.* w > 110 μm, h > 90 μm) except for the experiments testing the design conditions (Fig. 3a, Supplementary Figs 7 and 8). All the experiments were performed at a 5-μm immersion depth (unless it is stated otherwise) to satisfy the stable tweezers positioning conditions.

### Solution exchange

Depending on the height of the channels, the liquid volume inside the channel varied between 0.5 μl and 3 μl for different devices used in the experiments. The majority of the experiments (including molecular demonstrations) were performed in 1-μl channels. When we applied a vacuum using the pump at the outlet, a flow of 50 ± 5 μl min^−1^ was generated and kept running for 20 s to replace the solution inside the channel. The flow corresponded to 15 μl of solution insertion that was 5 times the channel volume for the biggest channel. The applied vacuum depended on the channel and side-opening size varying between couples of tens of mbar (~−10 to −30 mbar). Consequently, the liquid inside the channel could be exchanged within seconds (Supplementary Mov. 3).

### DNA capturing protocol

Molecular combing, one of the most commonly used techniques to stretch DNA, is based on a receding air-water interface to extend and align DNA molecules attached at one end to a solid surface^34^. Using molecular combing, DNA bundles can be stretched and assembled between micromachined structures^35^,^36^,^37^. Electrophoretic stretching is another technique to stretch DNA molecules between two micromachined structures^38^,^39^. We combined these two techniques, dielectrophoresis (DEP)-assisted lateral combing, to capture a λ-phage DNA (Takara Bio Inc.) bundle bridging between tweezers tips (Fig. 4a). A drop (5 μl) of λ-phage DNA solution (0.175 mg ml^−1^ DNA, 5 mM Tris and 0.5 mM EDTA) was placed on the PDMS device. Using the automated XYZ stage, tweezers tips (with aluminum coating) were placed inside the droplet while applying an AC voltage (1 MHz, 3.2–4.8 V_p-p_) between the tweezers tips (gap varying between 8–12 μm). DNA molecules could attach randomly on the aluminum coating of the tips. Then, the microfluidic device was moved laterally (Supplementary Fig. 10) allowing one of the tips to leave the droplet. As DNA molecules were attached on the tip surface, receding air-water interface extended the DNA molecules forming a DNA bundle drawn out of the droplet. When the second tip left the droplet, the DNA molecules attached on the aluminum coating of the second tip. As a result, a bundle was formed and captured between the two tips of the tweezers (Supplementary Mov. 1). We could capture a DNA bundle even without applying any voltage for DEP. However, the DEP-assisted capturing was more efficient even for lower concentration DNA solutions.

### Experimental process

The developed software (using LabVIEW) was capable of controlling the XYZ stage, microfluidic pump, lock-in-amplifier, heater, temperature and humidity sensors, a camera and additional function generators (in case necessary, *e.g.* for DEP). As a result, the software could monitor the frequency response in real-time, position the microfluidic device, control the flow in the channel, apply DEP voltage, visualize the experiment and set the experimental conditions inside the box.

The fixed stage for the tweezers included a specially designed spring pin-connection system and necessary connections to the amplifiers and function generators. After the fabrication process, tweezers were wire bonded on a printed circuit board (PCB) designed to provide the appropriate connections to the equipment. Screwing the PCB on the fixed stage was enough to complete the electrical setup (<1 min). After connecting to the pump, the microfluidic device was placed on the XYZ stage and locked using two clippers at two sides of the device (<1 min).

After preparing the electrical and microfluidic setup, the first step was to fill the microfluidic device with solution by applying vacuum with the pump (usually <5 min depending on the length of the connection tubes). No air should be allowed in the channel or in the connection tubes. Then, the tweezers were placed manually under the brimming PDMS before running the insertion protocol for the tweezers. After detecting the air-liquid interface, the tweezers were kept at 50 μm away from the interface as the safe position (<5 min). For DNA capturing, microfluidic device was moved for the tweezers to enter the DNA droplet. After performing the DNA capturing protocol, the microfluidic device was positioned back to its initial position (<3 min). Therefore, the system was ready to perform experiments within 15 min.

## Additional Information

**How to cite this article**: Tarhan, M. C. *et al*. A rapid and practical technique for real-time monitoring of biomolecular interactions using mechanical responses of macromolecules. *Sci. Rep.* **6**, 28001; doi: 10.1038/srep28001 (2016).

## Supplementary Material

Supplementary Information

Supplementary Movie 1

Supplementary Movie 2

Supplementary Movie 3
