# Supplementary material for: A rapid and practical technique for real-time monitoring of biomolecular interactions using mechanical responses of macromolecules
Source: Sci Rep. 2016 Jun 16;6:28001. doi: 10.1038/srep28001 (PMC4910067; doi:10.1038/srep28001)
Supplement: Supplementary Information [file srep28001-s1.pdf]

A rapid and practical technique for real-time monitoring of biomolecular interactions using mechanical responses of macromolecules

Mehmet C. Tarhan[1,2,*], *Nicolas Lafitte*[2], *Yannick Tauran*[2,3], *Laurent Jalabert*[2], *Momoko Kumemura*[1], *Grégoire Perret*[2,4], *Beomjoon Kim*[1], *Anthony W. Coleman*[1,3], *Hiroyuki Fujita*[1] *and Dominique Collard*[1,2,*]

[1]CIRMM, Institute of Industrial Science, The University of Tokyo, Tokyo, Japan.

[2]LIMMS/CNRS-IIS UMI2820, Institute of Industrial Science, The University of Tokyo, Tokyo, Japan.

[3]LMI UMR5615, University of Lyon 1, Lyon, France.

[4]IEMN UMR8520, Lille, France.

Supplementary Figures

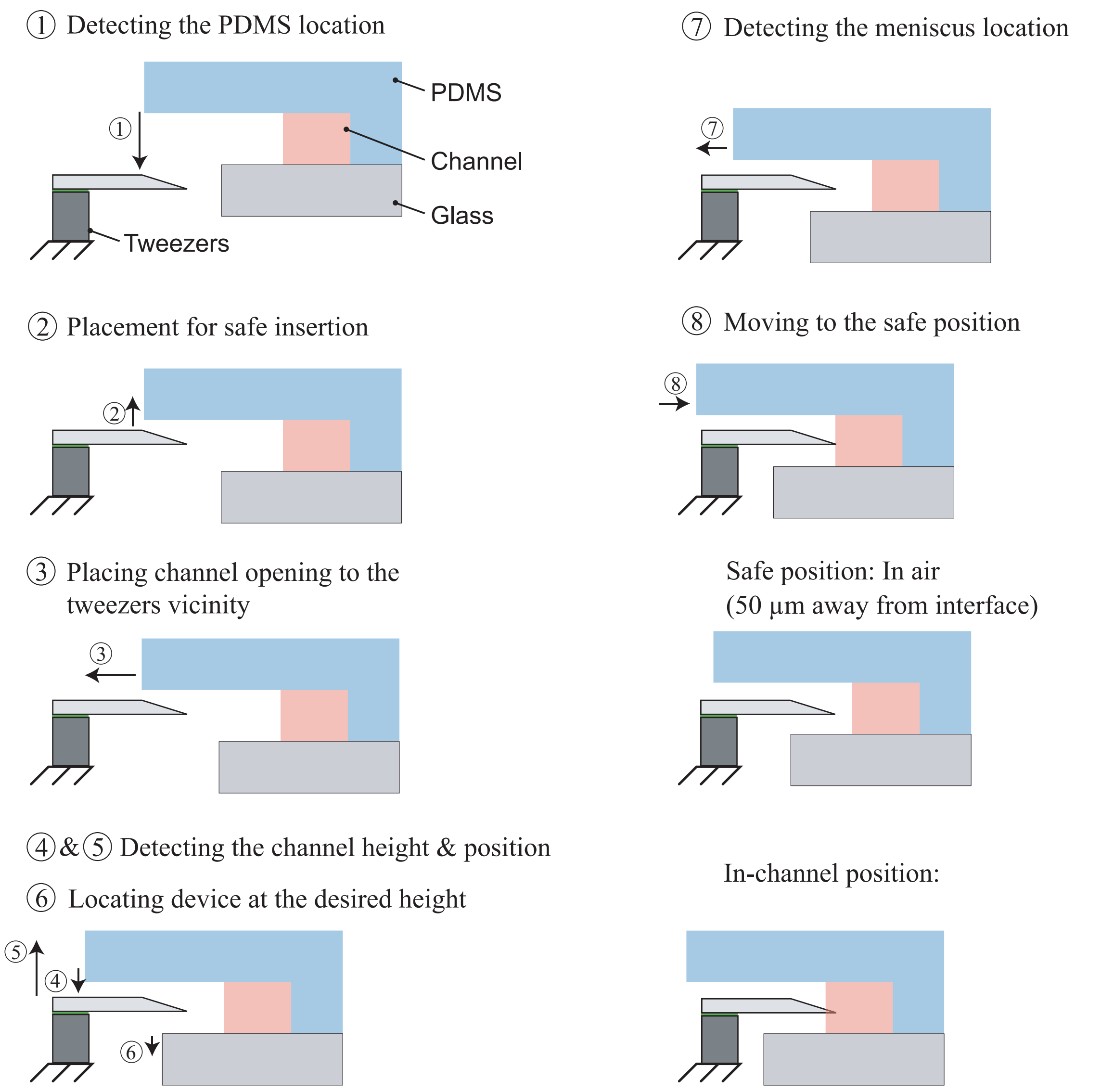

Supplementary Figure 1: Tweezers insertion protocol was performed in three steps. i) At first the PDMS rim was used to detect the top-level of the channel (1,2) and tweezers were positioned close to the channel vicinity (3). ii) The second step was to detect the precise location of the channel. The top and bottom level of the channel were detected (4,5) and positioned at the required height (6). iii) The last step was to detect the air-liquid meniscus (7). After detection, a safe position was saved before DNA capturing (8). Assays were performed inside channel at the desired immersion depth.

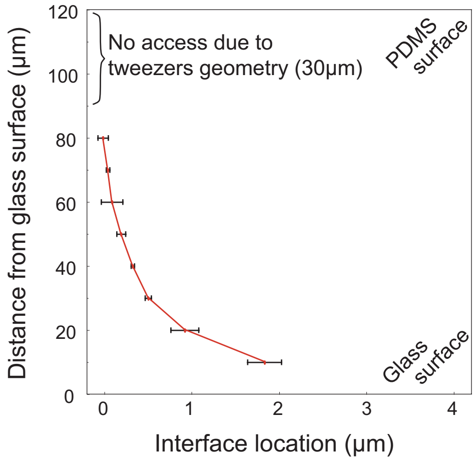

Supplementary Figure 2: The position of the air-liquid interface changes slightly closer to the glass surface due to the hydrophilic surface. 30 µm above the glass surface resulted in <0.5 µm changes in the air-liquid interface position. Error bars correspond to standard deviations (n>5 for all cases).

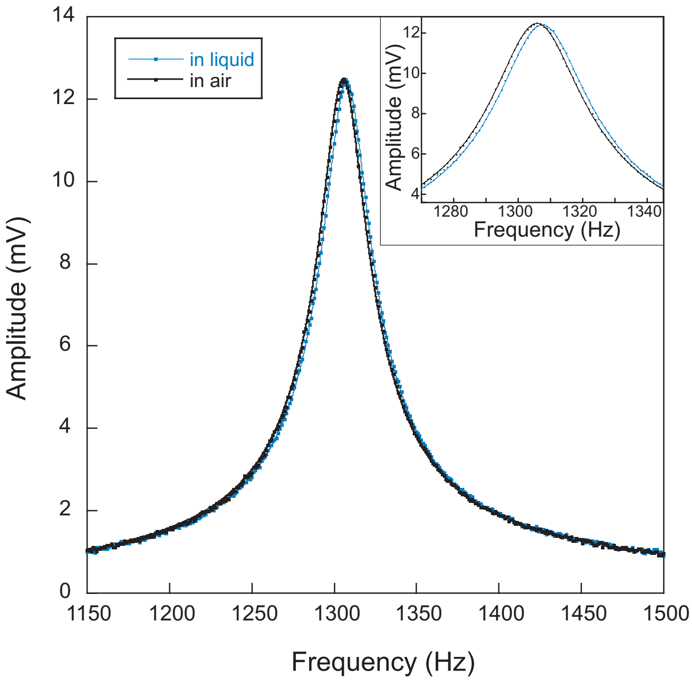

Supplementary Figure 3: An example of tweezers' frequency response in air and in liquid without any macromolecules between the tips. Inserting the SNT tips in the liquid resulted in 1.6 Hz increase in f_R and 0.5% decrease in A_{max} due to the air-liquid interface.

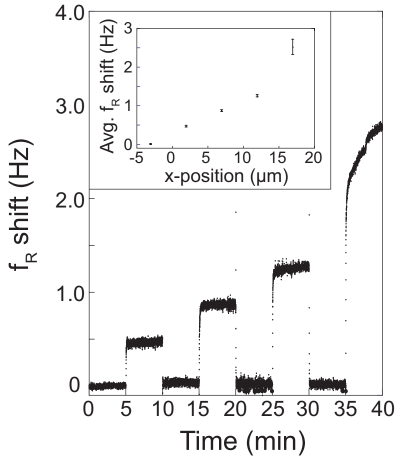

Supplementary Figure 4: Tweezers were inserted into the channel (from an opening of h= 115, w= 108 µm) at different immersion depth. Between each step, tweezers were taken out to a position 3 µm away from the air-liquid interface. The position of each step with respect to the air-liquid interface is shown in the inset. The error bars correspond to standard deviations.

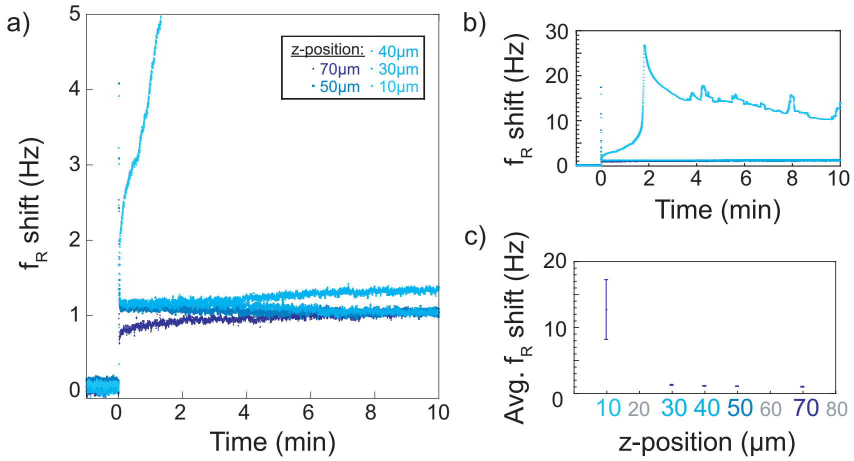

Supplementary Figure 5: a) Tweezers were inserted into channel (t=0 min) at different insertion height (varying from 10 µm to 70 µm above the glass surface for a device with h=115 µm, w=108 µm). b) Zoom out view of the results shown in (a). The initial immersion depth was 5 µm for all the cases. However, insertion at 10-µm height caused solution to go out under the tweezers because of the capillary effect between the glass surface and the bottom of the tweezers. c) A graph showing the average resonance frequency shift. Z-position corresponds to the distance from the glass surface. The error bars correspond to standard deviations.

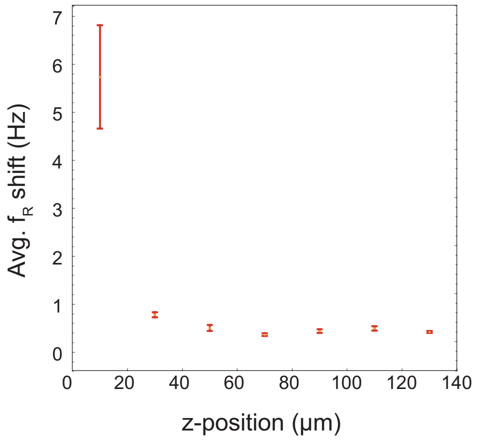

Supplementary Figure 6: Another example of devices with larger openings (h=171 µm, w= 134 µm) showing similar characteristics as supplementary figure 4. Tweezers insertion at 10-µm height resulted in an unstable response. Height > 30 µm showed stable results. Z-position corresponds to the distance from the glass surface. The error bars correspond to standard deviations.

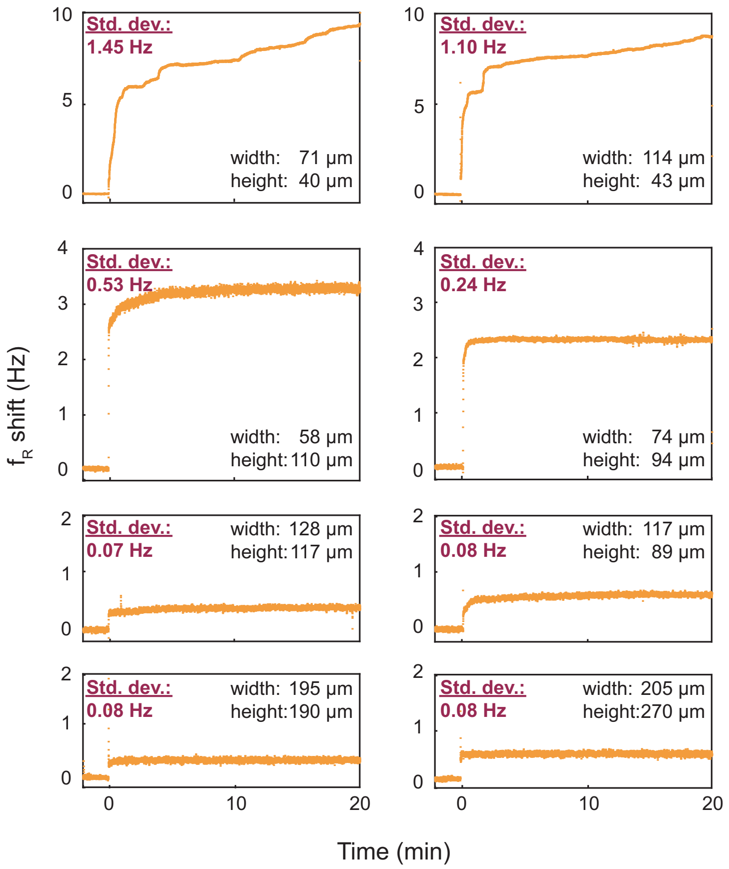

Supplementary Figure 7: Dimensions of the side opening affected the stability. Tweezers were inserted into the channel (at t = 0 min) and monitored for 20 min for stability. Some of the examples are shown above. Lower openings caused instability due to capillary effect between the bottom of the tweezers and the glass surface as seen in the given standard deviations. Similarly, narrow openings caused low stability due to the capillary effect between the tweezers arms and the PDMS walls.

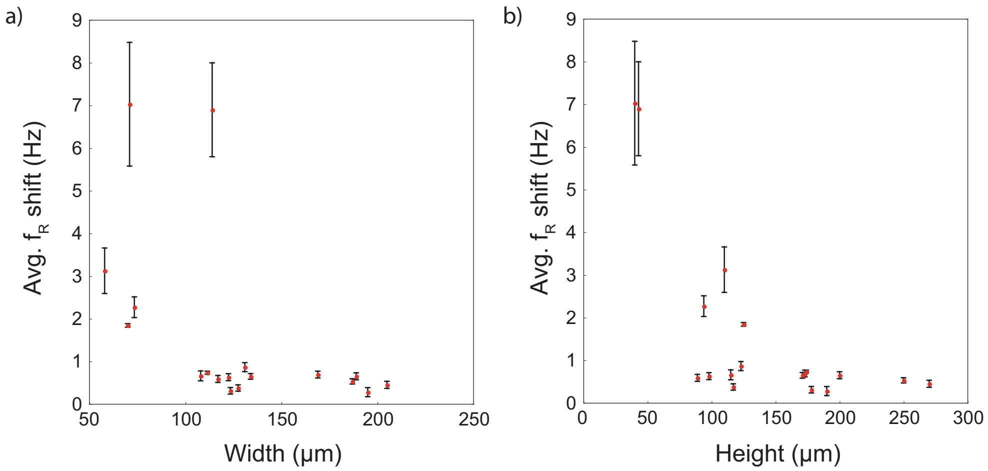

Supplementary Figure 8: Devices with different size opening were monitored for stability based on a) the width and b) the height of the opening. Some examples of the monitored devices were shown in supplementary figure 1. Wider (w>110 μm) and higher openings (h>90 μm) provided more stable results.

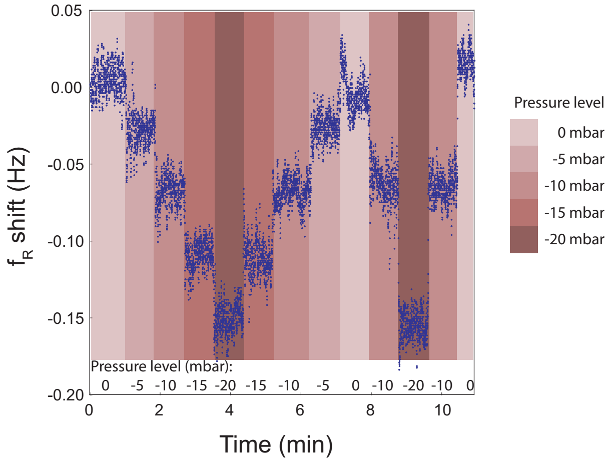

Supplementary Figure 9: Pump working at the withdrawn mode generated a flow in the channel. Applying different pressure did not compromise stability. In the experiments, the necessary pressure level was applied (for 20 s) to induce a flow rate of ~50 µl min^{-1}.

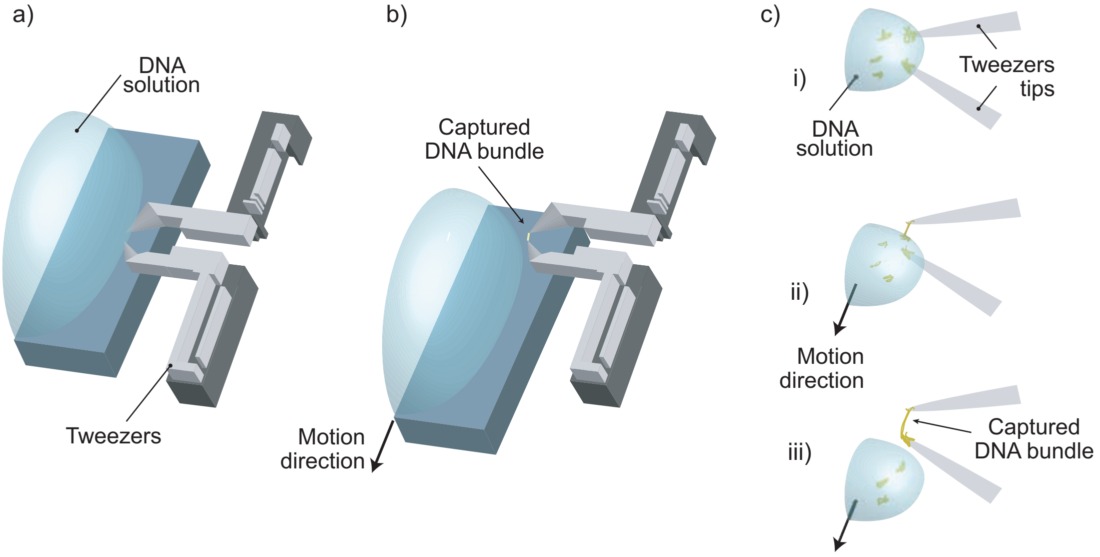

Supplementary Figure 10: DNA capturing was performed using DEP-assisted lateral combing. a) Tweezers were inserted into DNA solution (0.175 µg ml^{-1}). Then an AC voltage (1 MHz, 3.2-4.8 V_{p-p}) was applied between tweezers tips. b) The stage was moved laterally to remove the tips of the tweezers consequently for the capturing process. c) Molecular combing steps: (i) Parts of DNA molecules were attached on the Al-coated tips after inserted into the solution. (ii) Lateral motion of the droplet allowed one of the tips to be removed from the droplet while performing molecular combing. Regions of DNA drawn out of the solution were stretched and aligned. (iii) The bridge was formed with stretched and aligned DNA molecules when the second tip was removed from the droplet.

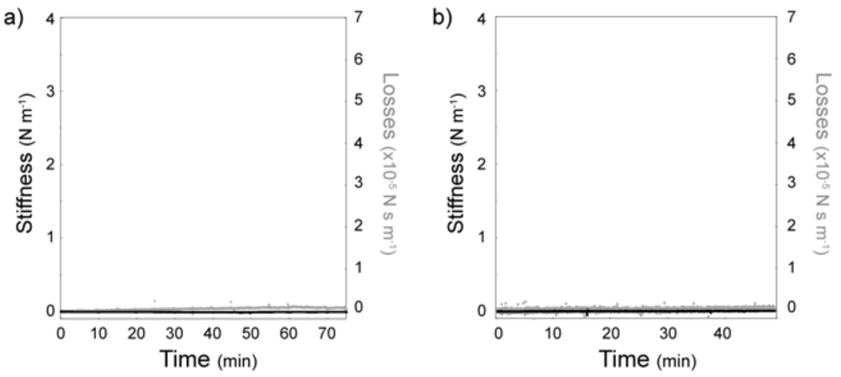

Supplementary Figure 11: No-DNA control experiments did not show any increase in the stiffness or the viscous losses for a) decreasing pH (for 5 min of buffer/acid cycles with a pH decreasing from 4.8 to 2.1) and b) increasing Ag concentration (from 10 µM to 100 mM). For better comparison both axis are having the same scales as the DNA experiments shown in Fig. 5.

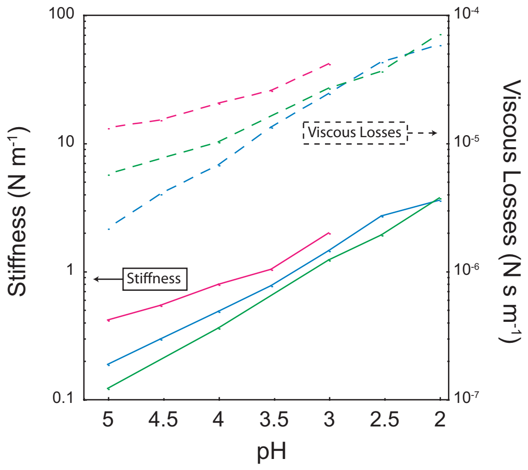

Supplementary Figure 12: Different DNA bundles resulted in similar characteristics when exposed to varying pH levels. Decreasing pH resulted in increasing stiffness and viscous losses.

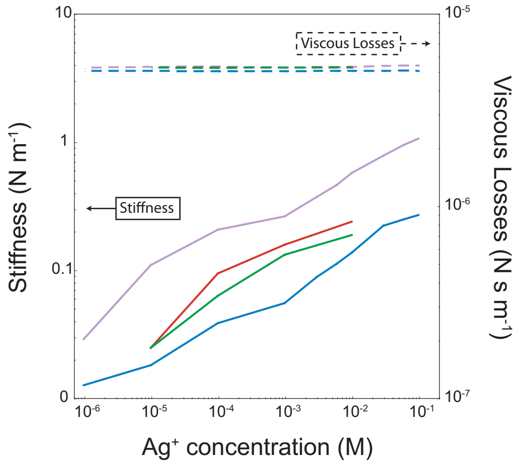

Supplementary Figure 13: Different DNA bundles resulted in similar characteristics when exposed to increasing Ag^{+} concentration. Unlike the pH case, increasing Ag^{+} concentration affected only the stiffness. Viscous losses stayed constant even though stiffness increased with the increasing Ag^{+} concentration.

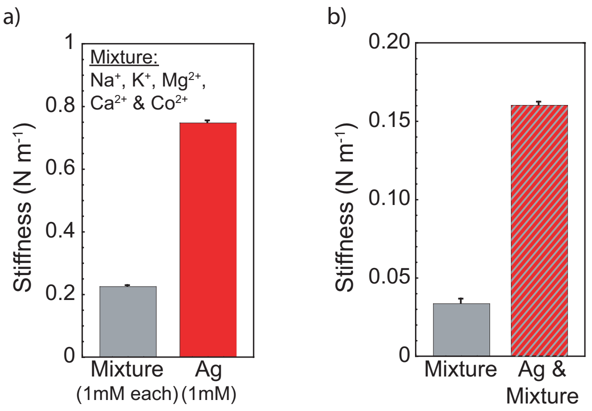

Supplementary Figure 14: Ag^+ selectively and specifically affected the DNA bundle. a) A DNA bundle was exposed to 1 mM Ag^+ solution right after a metal ion mixture (Na^+, K^+, Mg^{2+}, Ca^{2+} and Co^{2+}, 1 mM each). The stiffness showed a big increase even though the pH of solutions were almost identical (4.85 and 4.80 respectively). b) A similar result was obtained when the metal mixture was followed by an Ag-metal-mixture (Ag^+, Na^+, K^+, Mg^{2+}, Ca^{2+} and Co^{2+}, 1 mM each). Ag^+ selectively showed an effect on the DNA bundle in presence of other metal ions.

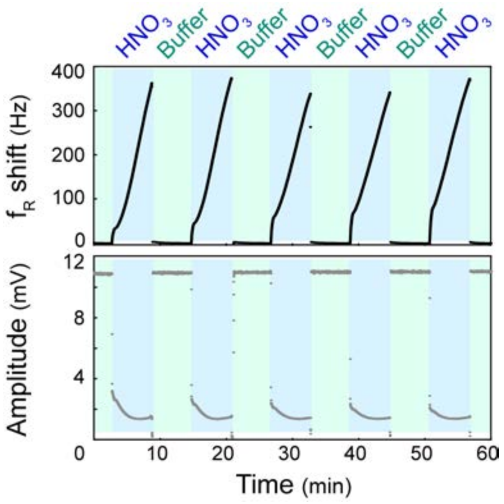

Supplementary Figure 15: Effect of acid on the mechanical properties of the DNA bundle was reversible even at very low pH. Tris buffer (pH 6.8) followed HNO_3 solution (pH 2.1) injection in 5 min cycles. HNO_3 solution increased f_R and decreased A_{max} of the tweezers and buffer returned them back to the initial values.

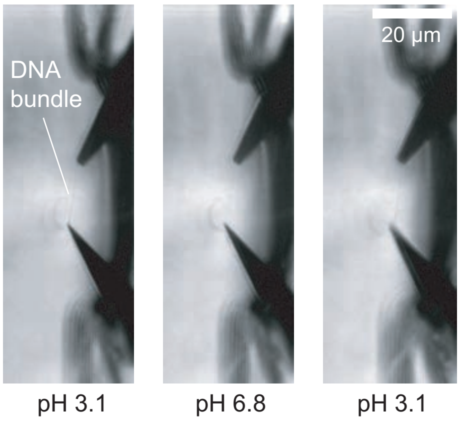

Supplementary Figure 16: Images of DNA bundle at different pH levels monitored with visual light. At low pH level, (-) charges on DNA molecules possibly decreased due to protonation and thus, DNA bundle became tighter causing an increase in the viscous losses (image on the left). Increasing the pH resulted in deprotonation and thus DNA molecules in the bundle started repelling each other (image in the middle). Therefore, the bundle was difficult to visualize. 3 minutes after acid injection, the bundle was visible again (image on the right). The differential interference contrast (DIC) images were taken with a photometrics camera (Cascade II) on an inverted microscope stage (Olympus IX71).

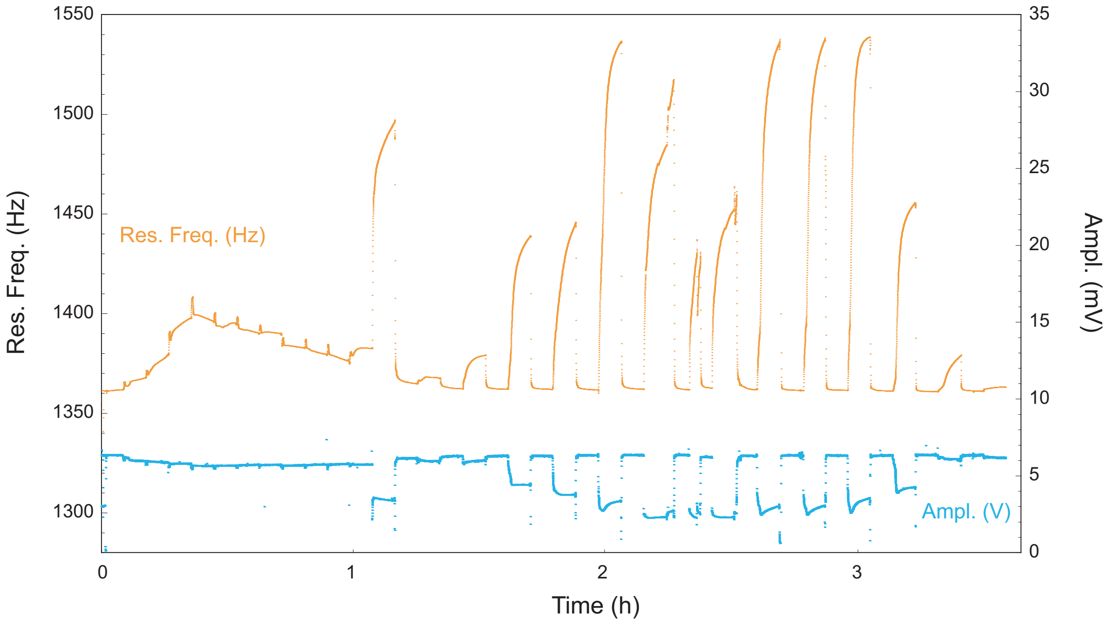

Supplementary Figure 17: Longer experiments (> 3h) were also performed on the same DNA bundle. The example shown in this figure examined nickel ion effect on DNA (until t = 1 h). Then, on the same DNA, zinc ion effect at low pH values were tested for demonstration purposes.

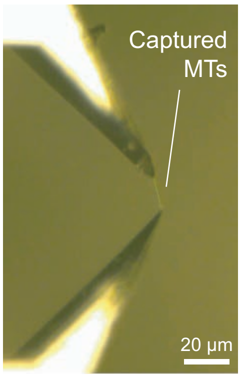

Supplementary Figure 18: Microtubules were captured between the tweezers tips to demonstrate possibility of capturing other macromolecules using the proposed method. The gap between the tips is 16 µm.

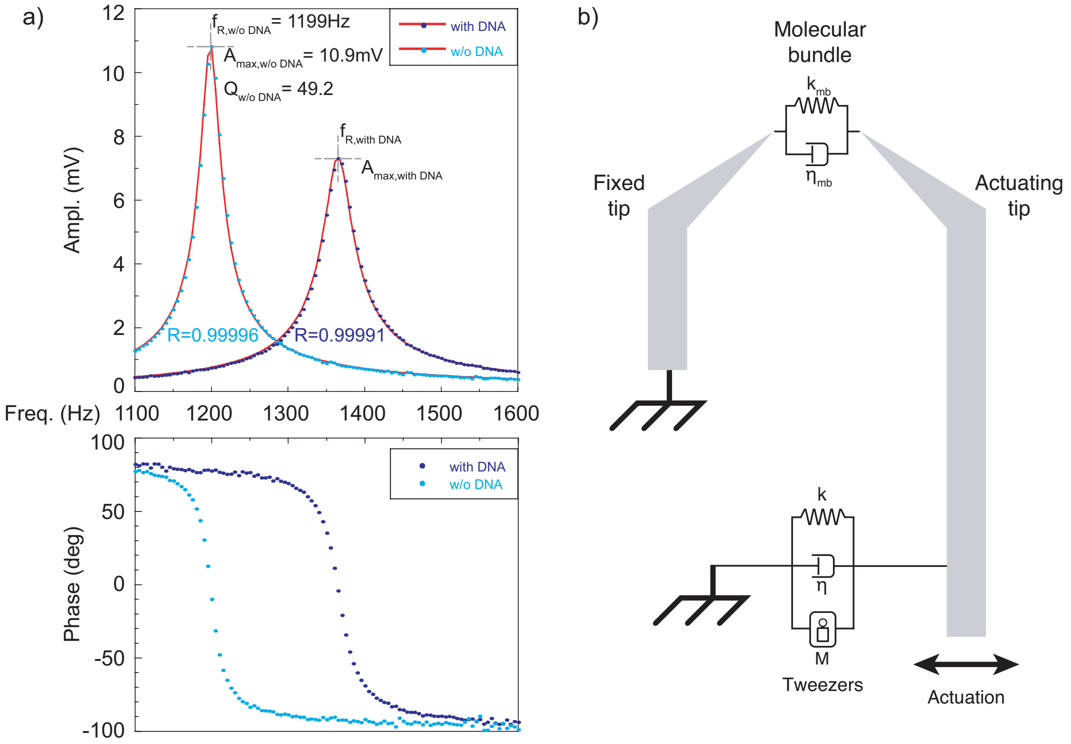

Supplementary Figure 19: a) Frequency response (amplitude and phase) of tweezers with and without DNA (in air). Blue dots correspond to the measurements and red lines correspond to the performed fitting by least square method to extract the f_R and A_{max} values. b) A damped harmonic oscillator model was used to derive the molecular stiffness and viscous losses values of the molecular bundle.

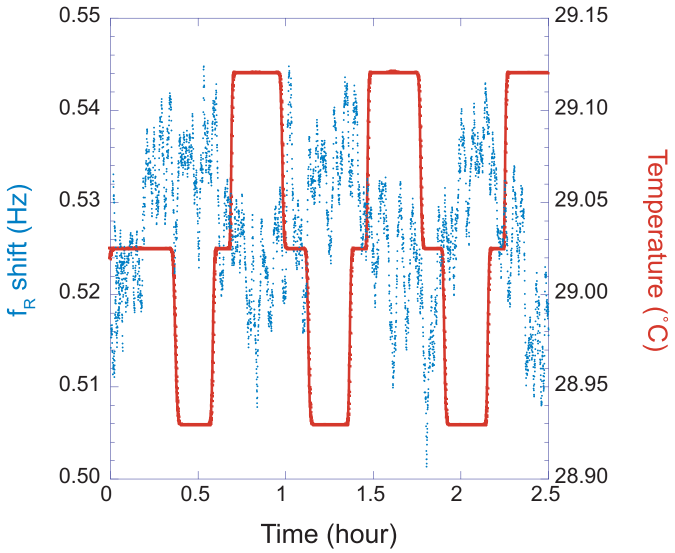

Supplementary Figure 20: A portion of the raw data presented in figure 2a. After smoothing the correlation between the f_R and temperature could be seen much more clearly. Temperature increase of 0.2°C inside the box caused a decrease of ~0.02 Hz in f_R

Supplementary Movies

Supplementary Movie 1: DNA capturing

Supplementary Movie 2: Automated detection of air-liquid interface

Supplementary Movie 3: Liquid exchange in the microfluidic device
